# Supplementary material for: Conditional chemoconnectomics (cCCTomics) as a strategy for efficient and conditional targeting of chemical transmission
Source: eLife. 2024 Apr 30;12:RP91927. doi: 10.7554/eLife.91927 (PMC11060718; doi:10.7554/eLife.91927)
Supplement: Figure 3—source data 3. [file elife-91927-fig3-data3.zip › Figure 3 source data3 part 1.zip!/Figure 3-Source data 3-part1/1071f3-1-CRISPResso_Report_jvi77nmx.zip!/CRISPResso_on_1071f3_2/9.Alleles_frequency_table_around_sgRNA_actactacatctccgtggag.pdf]

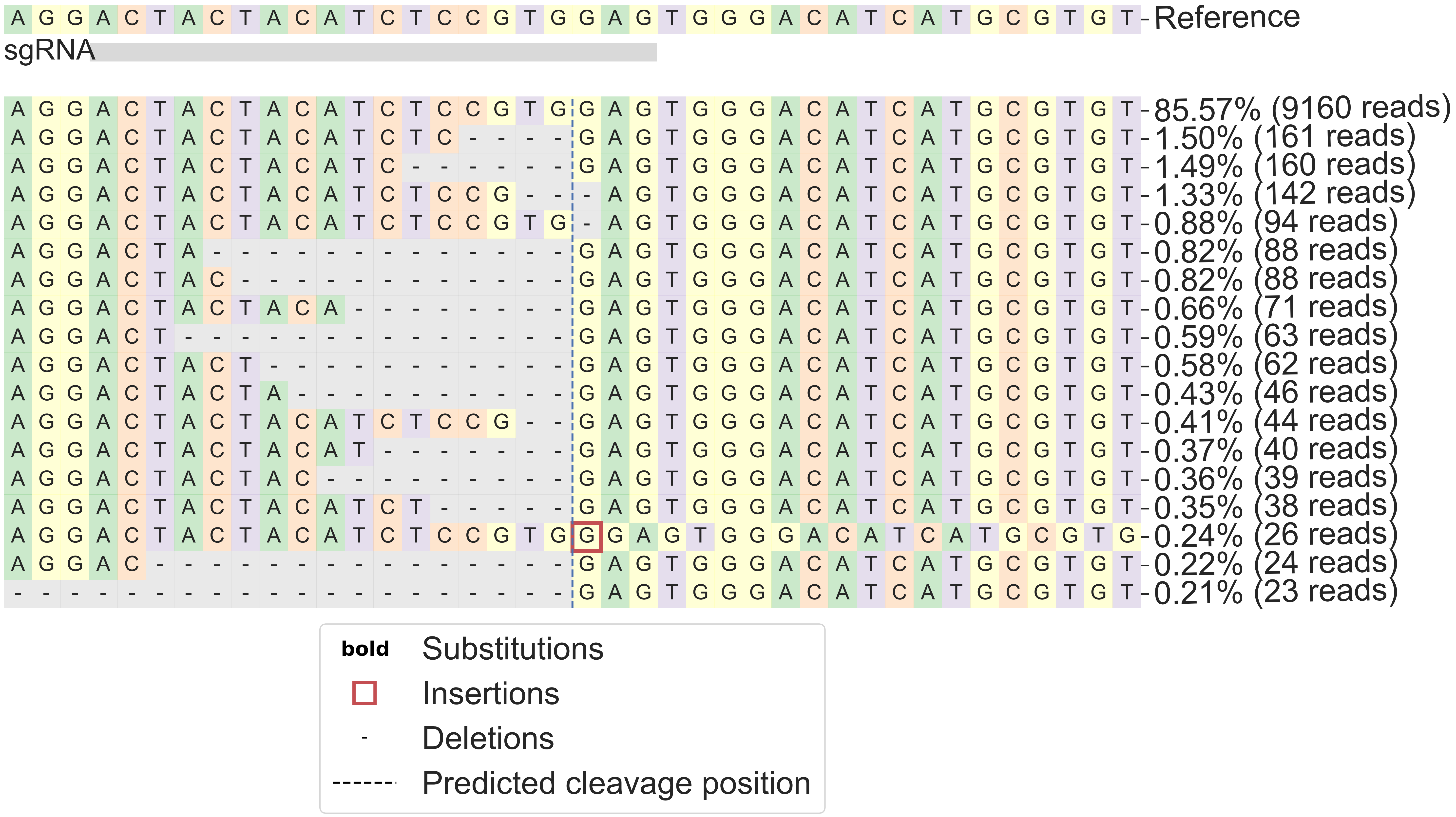
AGGACTACTACATCTCCGTGGAGTGGACATCATGCGTGT - Reference
sgRNA
AGGACTACTACATCTCCGTGGAGTGGACATCATGCGTGT - 85.57% (9160 reads)
AGGACTACTACATCTC----GAGTGGACATCATGCGTGT - 1.50% (161 reads)
AGGACTACTACATC------GAGTGGACATCATGCGTGT - 1.49% (160 reads)
AGGACTACTACATCTCCG---AGTGGACATCATGCGTGT - 1.33% (142 reads)
AGGACTACTACATCTCCGTG-AGTGGACATCATGCGTGT - 0.88% (94 reads)
AGGACTA-------------GAGTGGACATCATGCGTGT - 0.82% (88 reads)
AGGACTAC------------GAGTGGACATCATGCGTGT - 0.82% (88 reads)
AGGACTACTACA--------GAGTGGACATCATGCGTGT - 0.66% (71 reads)
AGGACT--------------GAGTGGACATCATGCGTGT - 0.59% (63 reads)
AGGACTACT-----------GAGTGGACATCATGCGTGT - 0.58% (62 reads)
AGGACTACTA----------GAGTGGACATCATGCGTGT - 0.43% (46 reads)
AGGACTACTACATCTCCG--GAGTGGACATCATGCGTGT - 0.41% (44 reads)
AGGACTACTACAT-------GAGTGGACATCATGCGTGT - 0.37% (40 reads)
AGGACTACTAC---------GAGTGGACATCATGCGTGT - 0.36% (39 reads)
AGGACTACTACATCT-----GAGTGGACATCATGCGTGT - 0.35% (38 reads)
AGGACTACTACATCTCCGTGGGAGTGGACATCATGCGTG - 0.24% (26 reads)
AGGAC---------------GAGTGGACATCATGCGTGT - 0.22% (24 reads)
--------------------GAGTGGACATCATGCGTGT - 0.21% (23 reads)
bold Substitutions
Insertions
- Deletions
Predicted cleavage position
